# Co-infection of broilers with *Ornithobacterium rhinotracheale* and H9N2 avian influenza virus

**DOI:** 10.1186/1746-6148-8-104

**Published:** 2012-07-02

**Authors:** Qing Pan, Aijing Liu, Faming Zhang, Yong Ling, Changbo Ou, Na Hou, Cheng He

**Affiliations:** 1 Key Lab of Animal Epidemiology and Zoonosis, Ministry of Agriculture, College of Veterinary Medicine, China Agricultural University, Beijing, 100193, China; 2 Beijing Veterinary Biological Manufactory, Beijing, 102600, China; 3 College of Veterinary Medicine, China Agricultural University, Yuanmingyuan Xilu, Haidian District, Beijing, 100193, P.R. China

**Keywords:** Isolation, Characterisation, *Ornithobacterium rhinotracheale*, H9N2, Co-infect, Broiler

## Abstract

**Background:**

Since 2008, a progressive pneumonia has become prevalent in broilers and laying hens. This disease occurrs the first day after hatching and lasts more than 30 days, resulting in approximately 70% morbidity and 30% mortality in broilers. The objective of this study was to isolate and identify the pathogens that are responsible for the progressive pneumonia and establish an animal model for drug screening.

**Results:**

193 serum samples were collected from 8 intensive farms from 5 provinces in China and analysed in the current research. Our clinical survey showed that 65.2% to 100% of breeding broilers, breeding layers, broilers and laying hens were seropositive for ORT antibodies. From 8 intensive farms, six ORT isolates were identified by PCR and biochemical assays, and two H9N2 viruses were isolated. Newcastle Disease Virus (NDV) and Infectious BronchitisVirus (IBV) were excluded. Typical pneumonia and airsacculitis were observed both in broilers inoculated intraperitoneally with an ORT isolate alone and in those co-infected with ORT and H9N2 virus isolates. Specifically, the survival rate was 30%, 20%, 70%, 50% and 90% in birds inoculated with ORT+H9N2 virus, ORT followed by H9N2 virus, H9N2 virus followed by ORT, and ORT or H9N2 virus alone, respectively.

**Conclusions:**

The results of this study suggest that ORT infections of domestic poultry have been occurring frequently in China. ORT infection can induce higher economic losses and mortality if H9N2 AIV is also present. Although the isolation of ORT and H9N2 virus has been reported previously, there have been no reported co-infections of poultry with these two pathogens. This is the first report of co-infection of broilers with ORT and H9N2 virus, and this co-infection is probably associated with the outbreak of broiler airsacculitis in China, which has caused extensive economic losses.

## Background

*Ornithobacterium rhinotracheale*(ORT) causes respiratory infections, such as airsacculitis and pneumonia, in birds all over the world. ORT can be a primary or secondary etiological agent, depending on the strain virulence, environmental factors, the immune status of the host, and the presence of other infectious agents [1]. In recent years, outbreaks of respiratory disease associated with ORT have been reported all over the world, including the USA, France, Netherlands, Belgium, Spain, Germany, Hungary, Israel, Korea, Japan, Taiwan, Turkey, Brazil, Iran and South Africa [1-9]. This pathogen can cause economic losses in the poultry industry due to growth retardation and the designation of infected carcasses as unacceptable for human consumption [1,6,8]. The H9N2 avian influenza virus (AIV) is not only widespread in poultry, but it also has important implications for human health as a zoonotic infection [10,11]. Previous studies demonstrate that H9N2 virus infection contributes to respiratory distress and is involved in diseases caused by other respiratory pathogens in the poultry industry [12,13]. More recent reports indicate that two virulent Chinese isolates of H9N2 virus induced acute respiratory distress in mice [14], which are widely used in biomedical research, suggesting a potential public health risk.

Since 2008, progressive pneumonia has become prevalent in broilers and laying hens. This disease occurs on the first day after hatching and lasts for more than 30 days, resulting in approximately 70% morbidity and 30% mortality in broilers. In total, we isolated six ORT strains and ten H9N2 AIV strains from the field samples. Five of the six ORT strains were presented simultaneously with H9N2 and the other ORT was isolated from the breeder’s eggs. Although ORT and H9N2 have been isolated and identified separately in previous reports, there have been no reported co-infections of poultry with these two pathogens. In the current report, we present the characterisation of ORT and H9N2 isolates obtained from co-infected broilers, with the objective of understanding the aetiology of severe avian pneumonia.

## Methods

### Chickens and sampling procedures

In this study, a total of 291,000 chickens from 8 intensive farms in 5 provinces of China were evaluated, including 70,000 affected breeding broilers, 100,000 affected breeding layers, 95,000 broilers with pneumonia and 16,000 affected layers. In addition, 10,000 unaffected broilers were used as controls. In total, 193 serum samples were collected for the detection of antibodies against ORT using a commercial testing kit. The samples originated from Liaoning, Inner Mongolia, Hebei, Shandong and Beijing represented the ORT prevalence in Northern China.

### Antibody detection

Serum samples were tested for the presence of ORT antibodies using the FLOCKCHEK* *Ornithobacterium rhinotracheale* Antibody Test Kit (IDEXX GmbH, Switzerland) in accordance with the manufacturer’s instructions. All of the measurements were performed in duplicate, and the matching serum pairs were analysed on the same microtitre plate. The results were normalised using the positive and negative control sera provided in the kits and were expressed as the S/P value according to the following formula: $\text{S}/\text{P}=\left(\text{OD sample}-\text{OD negative control}\right)/\left(\text{OD positive control}-\text{OD negative control}\right)$. Sera with S/P values less than or equal to 0.4 were considered negative, and sera with S/P values greater than 0.4 were considered positive.

### Isolation and characterisation of ORT

The current study was approved by the Animal Care and Use Committee at China Agricultural University and was carried out in accredited animal biosafety level 3 facilities. The trachea and lungs were aseptically obtained from ORT serum-positive broilers. Streak cultures were performed using standard I nutrient agar (Merck, Germany) with 5% sheep blood and were incubated at 37°C under aerophilic conditions for 24–48 h. The positive colonies were identified by Gram stain and biochemical assays [1]. The biochemical tests assayed for oxidase, catalase, lysine, urea, indole, sulphuric acid (H_2_S), nitrate, gelatinase, motility, and carbohydrate fermentations, including glucose, mannose, lactose, sucrose, maltose, and galactose [2,7].

DNA samples were extracted from the positive ORT isolates using the DNeasy Tissue Kit (Qiagen, Germany) following the manufacturer’s instructions. The primers used in the study were designed based on the available gene sequence [1]. The forward primer was 5′- GAG AAT TAA TTT ACG GAT TAA G-3′, and the reverse primer was 5′-TTC GCT TGG TCT CCG AAG AT-3′. A 784-bp fragment of the 16 S rRNA was amplified and subjected to electrophoresis in a 1% (w/v) agarose gel. The PCR procedure included an initial incubation for 5 min at 94°C, 45 cycles for 30 s each at 94°C, annealing at 52°C for 60 s, and extension at 72°C for 90 s, with a final extension at 72°C for 7 min. The PCR product was sequenced, and the gene sequence was submitted to GenBank. The ORT isolates were designated by ORT/species/location/time.

### Isolation and characterisation of H9N2 AIV

Tissue samples, including lungs, pancreas and brain, were obtained aseptically from H9N2-positive broilers (detected by PCR). Approximately 100 mg of minced tissue was suspended in sterile physiological saline. Gentamycin (200 μg/ml) was added to the suspension. The undiluted supernatant (0.2 ml) was inoculated into 10-day-old specific-pathogen-free (SPF) chicken embryos. The eggs were candled daily, and the embryos that died within 24 h post inoculation (PI) were discarded. The allantoic fluid was collected, and virus was detected using a haemagglutination assay (HA). If no embryo death occurred, additional three blind passages were performed before designating any samples as negative. The antigenic characteristics of the virus subtype were determined by standard haemagglutination inhibition (HI), and the allantoic fluid containing virus was harvested and stored at −80°C until use [15].

The viral RNA was extracted from the allantoic fluid using the QIAamp viral RNA mini kit (Qiagen, Germany) in accordance with the manufacturer’s instruction. Reverse-transcription (RT) PCR was performed using specific primers [16]. The forward primer was 5′-CTY CAC ACA GAR CAC AAT GRR ATG-3′, and the reverse primer was 5′-GTC ACA CTT GTT GTT GTR TC-3′. The RT-PCR was performed in a 50 μl reaction mixture containing 10 μl of 5 × reaction buffer, 1 μl of mixed dNTPs, 1 μl of AMV enzyme, 2 μl of each primer, 4 μl of the RNA template, 2.5 μl of DTT, and 27.5 μl of distilled water. The PCR procedure was 94°C for 2 min, 30 cycles of 94°C for 1 min, 53°C for 1 min, 68°C for 1 min, and finally 68°C for 10 min. The PCR products were subjected to electrophoresis in a 1% (w/v) agarose gel and sequenced. The gene sequence was submitted to GenBank. The H9N2 virus isolate was designated by H9N2/species/location/time.

### Determination of the LD_50_ of ORT/chicken/Shandong/2011 and the ELD_50_ of H9N2/chicken/Shandong/2011

After single colony purification in 5% sheep blood agar, the positive colonies were grown in bouillon medium for 48 h at 37°C, and the number of colony formation units (CFU) in the culture was calculated post the inoculation.

Sixty 20-day-old healthy broilers were randomly assigned to six groups with 10 chickens per group and maintained in negative pressure isolators. The chickens were infected intraperitoneally with different dilutions of ORT/chicken/Shandong/2011 in 0.5 ml, including 10^0^, 10^-1^, 10^-2^, 10^-3^ and 10^-4^ cfu. Broilers inoculated intraperitoneally with sterile physiological saline served as a control group. Each group was observed daily for 14 days, and the LD_50_ was determined using the Reed-Muench method [17].

In another experiment, fifty 10-day-old SPF chicken embryos were randomly assigned to five groups with 10 eggs per group. Different dilutions of H9N2/chicken/Shandong/2011 (0.2 ml) were injected into the allantoic space of the embryos. The eggs were incubated in a humidified atmosphere (55%) at 37°C. The allantoic fluid was harvested 120 hPI, and the ELD_50_ was determined using the Reed-Muench method [17].

### Experimental infection with ORT/chicken/Shandong/2011 and H9N2/chicken/Shandong/2011

Sixty 21-day-old healthy broilers were randomly divided into six groups with 10 birds in each group. All of the birds were kept in negative pressure isolators. Group 1 was inoculated intraperitoneally with 10 LD_50_of ORT/chicken/Shandong/2011 in 0.5 ml, and at the same time, 100 ELD_50_ of H9N2/chicken/Shandong/2011 was administrated intranasally [12]. Group 2 received 10 LD_50_ of ORT/chicken/Shandong/2011 intraperitoneally in 0.5 ml and, three days later, received 100 ELD_50_ of H9N2/chicken/Shandong/2011 intranasally. Group 3 was inoculated intranasally with 100 ELD_50_ of H9N2/chicken/Shandong/2011 and, three days later, received 10 LD_50_ of ORT/chicken/Shandong/2011 intraperitoneally. Group 4 birds were inoculated intraperitoneally with 10 LD_50_ of ORT/chicken/Shandong/2011, and Group 5 was administered 100 ELD_50_ of H9N2/chicken/Shandong/2011 intranasally. Group 6 received an intraperitoneal injection of the sterile physiological saline as a negative control. Each group was observed daily, and all were sacrificed on day 14 PI. The broilers were euthanised by intraperitoneal injection of sodium pentobarbital. The gross lesions were inspected, and the main organs were collected for pathogen recovery.

## Results

### Clinical survey of the ORT antibody

A total of 291,000 birds, including 70,000 breeding broilers, 100,000 breeding layers, 95,000 broilers, 16,000 layers and 10,000 unaffected broilers were evaluated in this study. We totally collected 193 serum samples from the chickens for detection. Of the birds presenting with clinical signs, 127 of a total of 153 (83%) sera were positive and of apparently healthy birds 6 of 40 (15%) samples were seropositive (Table 1). With regards to age and seropositivity, the breeding species seroconverted at age 120 days to 280 days, the laying hens were around 120 days and the broilers were at day 32 to day 35 of age.

**Table 1:** The antibody detection in the field survey using the ORT test kit

**Species**	**Ages**	**No. stock**	**Samples**	**No. positive**	**Positivity (%)**
Breeding broilers	120-238	70,000	46	30	65.2^**^
Breeding layers	170-280	100,000	61	56	91.8^**^
Broilers	24-38	95,000	31	26	83.8^**^
Layers	120	16,000	15	15	100.0^**^
Total (infected)		281,000	153	127	83.0
Healthy broilers	32-35	10,000	40	6	15.0

^**^Significantly different from the healthy broilers (P<0.01).

### Isolation and characterisation of ORT/chicken/Shandong/2011

**Figure 1:**
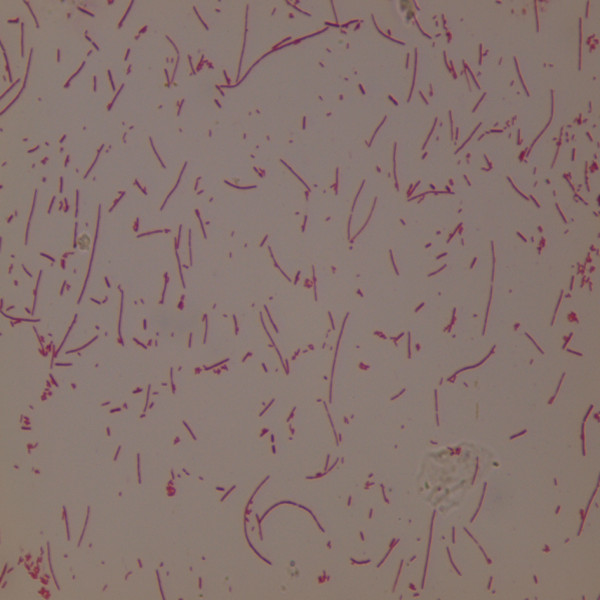
Gram stain of a solid culture of ORT/chicken/Shandong/2011 showing the pleomorphic shape of the isolate (Magnification × 1000).

ORT strain was successfully isolated from the lungs of diseased broilers by single colony purification. Subsequently, the isolate was classified using biochemical assays and a PCR assay. The strain was named ORT/chicken/Shandong/2011. The colony formed by ORT/chicken/Shandong/2011 was circular and small in size (1–3 mm in diameter), opaque to grey in colour and non-haemolytic in sheep blood agar. The ORT isolate was found to be Gram negative and multi-form upon microscopic inspection (Figure 1). Furthermore, ORT/chicken/Shandong/2011 showed the typical phenotypic traits of ORT (Table 2). The DNA extracted from the ORT/chicken/Shandong/2011 colony produced the expected 784-bp PCR product from the 16 S rRNA. The sequence of the 16 S rRNA was submitted to GenBank (accession number JN415768). A sequence analysis of the 16 S rRNA segment showed that the sequence from this isolate was 98%-100% homologous with the ORT reference strains (GenBank # HQ696786.1, U87100.1 and DQ860700.1).

**Table 2:** The biochemical properties of the two ORT isolates

**Tests**	**ORT/chicken/Shandong/2011**	**ORT/chicken/Inner Mongolia/2011**
Triple sugar iron agar	-	-
Motility	-	-
Oxidase	+	+
Catalase	-	-
Indole	-	-
Urease	+	+
Lysine decarboxylase	+	-
Nitrate	-	-
Gelatinase	-	-
Sucrose	-	-
Glucose	+	+
Galactose	+	+
Lactose	+	+
Maltose	+	+
Fructose	+	+

### H9N2/chicken/Shandong/2011 identification

The allantoic fluid of the inoculated embryos was positive by HA after the second passage of the virus in the SPF chicken embryos. The H9N2 virus was identified using a standard HI assay. Subsequently, the isolate was detected by RT-PCR, which yielded 468-bp amplicons. The gene sequence was submitted to GenBank (accession number JN566055). Corona virus-like particles were not detected in the allantoic fluid by RT-PCR. The sequence was 98%-100% homologous with H9N2 viruses, with accession numbers GU471873.1 and JF715009.1 in GenBank. In five severe cases, both ORT and H9N2 were isolated and identified in lungs from geographical distribution. Moreover, the presence of ORT was often identified while positive H9N2 isolates alone were confirmed in the diseased poultry with lower mortality.

### LD_50_ of ORT/chicken/Shandong/2011 and ELD_50_ of H9N2/chicken/Shandong/2011

After growing in medium for 48 h at 37°C, the concentration of ORT/chicken/Shandong/2011 was 2.49 × 10^9^ cfu/ml. The highest mortality occurred in group 1 and group 2 post inoculated with the ORT/chicken/Shandong/2011 strain (Table 3), and the LD_50_ of ORT/chicken/Shandong/2011 was determined to be 1.43 × 10^8^ cfu/ml in broilers. The ELD_50_ of H9N2/chicken/Shandong/2011 was 10^7.83^/ml (Table 4).

**Table 3:** **The determination of the LD**_**50**_**of the ORT/chicken/Shandong/2011 isolate**

**Group**	**No.**	**Dosage**	**Concentration**	**No. mortality**	**Mortality (%)**
1	10	0.5	2.49 × 10^9^ cfu/ml	10	10/10
2	10	0.5	2.49 × 10^8^ cfu/ml	6	6/10
3	10	0.5	2.49 × 10^7^ cfu/ml	1	1/10
4	10	0.5	2.49 × 10^6^ cfu/ml	0	0/10
5	10	0.5	2.49 × 10^5^ cfu/ml	0	0/10
6	10	0.5	control	0	0/10

**Table 4:** **The determination of the ELD**_**50**_**of the H9N2/chicken/Shandong/2011 isolate**

**Group**	**No.**	**Volumes**	**Dilutions**	**No. Mortality**	**Mortality (%)**
1	10	0.2	10^-6^	10	10/10
2	10	0.2	10^-7^	10	10/10
3	10	0.2	10^-8^	4	4/10
4	10	0.2	10^-9^	0	0/10
5	10	0.2	control	0	0/10

### Experimental infection with ORT/chicken/Shandong/2011 and H9N2/chicken/Shandong/2011

Some birds showed ruffled the feathers, inactivity and reduced appetite on day 2 PI with ORT and H9N2 virus simultaneously, ORT followed by H9N2 virus or ORT alone. By day 3 PI, most of the chickens abruptly showed clinical signs of respiratory disease, including respiratory distress, and exhibited more severe anorexia and emaciation. The infected birds died between days 3 and 5 PI. The survival rate was 30%, 20% and 50% in the ORT+H9N2 virus group, ORT/H9N2 virus group, and ORT group, respectively. In contrast, birds inoculated with H9N2 virus followed by ORT or the H9N2 virus alone displayed typical pneumonia for 4 days, and no mortality occurred within 3 days PI. The infected chickens died quickly after inoculation with ORT alone, and the survival rate was up to 70%, compared to 90% in the H9N2 group (Table 5). The infected birds displayed the typical lesions, such as fibrinous airsacculitis, pericarditis, peritonitis and scattered areas of haemorrhage in the lungs upon necropsy. No obvious gross lesions were observed in the liver and kidneys of the infected birds.

**Table 5:** Experimental infections with ORT/chicken/Shandong/2011 and H9N2/chicken/Shandong/2011

**Groups**	**No.**	**No. survival**	**Survival rate (%)**	**ORT recovery**	**H9N2 recovery**
1	10	3	30.0	3	3
2	10	2	20.0	2	2
3	10	7	70.0	7	7
4	10	5	50.0	5	5
5	10	9	90.0	9	9
6	10	10	100.0	0	0

Indications:

Group 1 chickens were inoculated intraperitoneally with 10 LD_50_ of ORT/chicken/Shandong/2011 in 0.5 ml, and 100 ELD_50_ of H9N2/chicken/Shandong/2011 was administrated intranasallyin 0.2 ml at the same time.

Group 2 chickens were given 10 LD_50_ of ORT/chicken/Shandong/2011intraperitoneally in 0.5 ml and received 100 ELD_50_ of H9N2/chicken/Shandong/2011intranasallyin 0.2 ml three days later.

Group 3 birds were inoculated intranasally with 100 ELD_50_ of H9N2/chicken/Shandong/2011in 0.2 ml and three days later received intraperitoneally with 10 LD_50_ of ORT/chicken/Shandong/2011in 0.5 ml.

Group 4 birds were inoculated intraperitoneally with 10 LD_50_ of ORT/chicken/Shandong/2011in 0.5 ml.

Group 5 chickens were given 100 ELD_50_ of H9N2/chicken/Shandong/2011intranasally in 0.2 ml.

Group 6 birds received the sterile physiological saline intraperitoneally as a negative controlin 0.5 ml.

## Discussion

In the current study, 83.0% of clinical blood samples from poultry farms were positive for ORT antibodies by ELISA. The ORT isolated from the infected lungs of a 32-day-old broiler grew on sheep blood agar, and the H9N2 virus was isolated from the same broiler and identified by HI and RT-PCR. Based on the sequence analysis, the Chinese ORT isolate is 98.0 to 100% homologous to other ORT isolates in GenBank. Broilers inoculated intraperitoneally with ORT/chicken/Shandong/2011 alone displayed pneumonia and typical airsacculitis, and co-infection of the broilers with ORT and H9N2 virus isolates induced higher mortality than infection with ORT or H9N2 virus alone. Therefore, these results satisfy Koch’s postulates for confirming the role of a suspected bacterial pathogen in disease. The results of this study strongly suggest that co-infection with ORT and H9N2 virus is responsible for the current severe pneumonia with high mortality in broilers of China.

In our clinical setting, ORT was associated with 20-30% and 10-20% mortality in broilers and layers, respectively. However, the birds infected with the ORT isolated in this study had a 50% mortality rate, and a co-infection with the H9N2 virus resulted in 70% death. Our findings suggest that primary infection with ORT might play a major role in the development of severe pneumonia, and secondary infection with H9N2 further increases the mortality. These findings are different from previous reports in which no pneumonia or airsacculitis was induced by aerosol, intra-tracheal or intra-thoracic inoculation with ORT alone [18]. Only intravenous inoculation has been reported to induce clinical signs in SPF chickens, with at most 20% mortality [19], and no airsacculitis has been previously seen in the field. Given these conflicting results, it is uncertain if ORT should be regarded as a primary pathogen [1]. Additionally, it is generally acknowledged that ORT and *Avian pneumovirusvirus* (APV) infections synergistically aggravate respiratory symptoms in turkeys. An aerosol inoculation of ORT without a viral primer did not result in lesions [20,21]. Furthermore, viral agents could trigger higher mortality independent of ORT infection, such as NDV [1] and APV [21,22]. A recent report confirmed that IBV and *E. coli* infection exacerbated ORT pathogenesis in adult laying hens [23]. However, none of the above-mentioned viruses were identified in the present study. Interestingly, the birds infected with ORT alone developed an exudative pneumonia and extensive haemorrhage in the lungs and kidneys. This pattern of pathology is not consistent with previous reports. These histological results indicate that this newly isolated ORT is different from formerly reported ORT serotypes [20-22,24]. Because the serotype of the ORT isolated in this study is unclear, further investigation is needed to identify its specific serotype and characterise ORT pathogenesis based on serotype. After inoculation of broilers with ORT and H9N2 virus together, widespread haemorrhage and fibrosis in the respiratory tract were the most notable features of the infection, which led to occlusion of the air capillaries, respiratory distress and the increased mortality. These histopathological lesions are analogous to those described in birds late during the course of avian influenza H9N2 infection alone [11,14]. Notably, the severe pulmonary fibrosis was observed in the animals inoculated first with ORT followed by the H9N2 virus. The clinical signs and the respiratory lesions observed during necropsy of the birds infected with the H9N2+ORT combination and the H9N2 group confirm that ORT, not a viral agent, triggers the overt respiratory symptoms.

Our findings are also different from previous reports in which ORT could be involved in infections with *E. coli O2:K1*[22], *Bordetella avium*[25], and *Chlamydophila psittaci*[26] as well as a combination of *E. coli* and APV [22]. In our pilot study (unpublished data), *E. coli* and *Chlamydophila psittaci* were occasionally identified in the latter phase of the ORT infection. However, co-infection of ORT and *E. coli* or *Chlamydophila psittaci* could not reproduce the typical pathology of the pneumonia and airsacculitis, such as clots of fibrin in the air sacs and haemorrhage in lungs. In the co-infection, ORT may dominate the primary infection, followed by secondary bacterial infections. ORT was isolated and identified in birds with clinical signs immediately after hatching in the previous reports [24]. In current study, ORT/chicken/Liaoning/2010 was isolated from the egg yolks of the breeder’s eggs, suggesting the possibility of vertical transmission from parents to offspring.

## Conclusion

Our data show that ORT infections have been occurring frequently in China. The current experimental study confirmed that ORT infection alone could induce high mortality. Moreover, ORT infection could produce a higher level of mortality and economic loss if H9N2 AIV was also present. Although ORT and the H9N2 virus have been isolated and identified separately in previous reports, this is the first report of co-infection of broilers with ORT and H9N2 AIV, and this co-infection might be probably associated with the outbreak of broiler airsacculitis in China, which caused an extensive economic loss. Therefore, further investigation of the resistance and pathogenesis of ORT is urgently needed.

## Abbreviations

ORT, Ornithobacterium rhinotracheale; AIV, Avian influenza virus; HA, Haemagglutination assay; HI, Haemagglutination inhibition; IBV, Infectious bronchitis virus; NDV, Newcastle disease virus; ELISA, Enzyme-linked immunosorbent assay; PCR, Polymerase chain reaction; CFU, Colony formation units; PI, Post inoculation; SPF, Specific pathogen free.

## Authors’ contributions

QP contributed to the study design, evaluated the data, and drafted and wrote the manuscript. AL isolated and characterised the ORT. FZ isolated and characterised the AIV H9N2. YL collected the samples and helped contact the chicken farmers. CO performed the statistical analyses. NH helped with the ELISA.CH contributed to the study design, obtained the funding, and evaluated the microarray. All authors have read and approved the final manuscript.
